# Decompression alone versus decompression with instrumental fusion the NORDSTEN degenerative spondylolisthesis trial (NORDSTEN-DS); study protocol for a randomized controlled trial

**DOI:** 10.1186/s12891-018-2384-0

**Published:** 2019-01-05

**Authors:** Ivar Magne Austevoll, Erland Hermansen, Morten Fagerland, Frode Rekeland, Tore Solberg, Kjersti Storheim, Jens Ivar Brox, Greger Lønne, Kari Indrekvam, Jørn Aaen, Oliver Grundnes, Christian Hellum

**Affiliations:** 1 Kysthospitalet in Hagevik, Orthopedic Clinic, Haukeland University Hospital, Hagavik, N- 5217 Bergen, Norway; 2 Department of Clinical Medicine, University of Bergen, N- 5007 Bergen, Norway; 3 Department of Orthopedic Surgery, Ålesund Hospital, Møre and Romsdal Hospital Trust, N-6026 Ålesund, Norway; 4 Oslo Centre for Biostatistics and Epidemiology, Research Support Services, Oslo University Hospital, N-0424 Oslo, Norway; 5 Department of Neurosurgery, University Hospital of Northern Norway, N-9019 Tromsø, Norway; 6 Department of Clinical Medicine, University of Tromsø - The Arctic University of Norway, N-9019 Tromsø, Norway; 7 The Norwegian Registry for Spine Surgery (NORspine), Northern Norway Regional Health Authority, N-9038 Tromsø, Bodø Norway; 8 Research and Communication Unit for Musculoskeletal Health (FORMI), Oslo University Hospital, N-0424 Oslo, Oslo Norway; 9 Department of Orthopedics, Akershus University Hospital, N-1474 Lørenskog, Oslo Norway; 10 Department of Physical Medicine and Rehabilitation, Oslo University Hospital, N-0424 Oslo, Norway; 11 Department of Research, Levanger Hospital, Nord-Trøndelag Hospital Trust, N-7600 Levanger, Norway; 12 Department of Orthopedic Surgery, Innlandet Hospital Trust, N-2609 Lillehammer, Lillehammer Norway; 13 Division of Orthopaedic Surgery, Oslo University Hospital, N-0424 Oslo, Norway

**Keywords:** Spinal stenosis, Degenerative spondylolisthesis, Randomized controlled trial, Decompression, Fusion, Clinical outcomes, NORDSTEN

## Abstract

**Background:**

Fusion in addition to decompression has become the standard treatment for lumbar spinal stenosis with degenerative spondylolisthesis (DS). The evidence for performing fusion among these patients is conflicting and there is a need for further investigation through studies of high quality. The present protocol describes an ongoing study with the primary aim of comparing the outcome between decompression alone and decompression with instrumented fusion. The secondary aim is to investigate whether predictors can be used to choose the best treatment for an individual. The trial, named the NORDSTEN-DS trial, is one of three studies in the Norwegian Degenerative Spinal Stenosis (NORDSTEN) study.

**Methods:**

The NORDSTEN-DS trial is a block-randomized, controlled, multicenter, non-inferiority study with two parallel groups. The surgeons at the 15 participating hospitals decide whether a patient is eligible or not according to the inclusion and exclusion criteria. Participating patients are randomized to either a midline preserving decompression or a decompression followed by an instrumental fusion.

Primary endpoint is the percentage of patients with an improvement in Oswestry Disability Index version 2.0 of more than 30% from baseline to 2-year follow-up. Secondary outcome measurements are the Zürich Claudication Questionnaire, Numeric Rating Scale for back and leg pain, Euroqol 5 dimensions questionnaire, Global perceived effect scale, complications and several radiological parameters. Analysis and interpretation of results will also be conducted after 5 and 10 years.

**Conclusion:**

The NORDSTEN/DS trial has the potential to provide Level 1 evidence of whether decompression alone should be advocated as the preferred method or not. Further on the study will investigate whether predictors exist and if they can be used to make the appropriate choice for surgical treatment for this patient group.

**Trial registration:**

ClinicalTrials.gov Identifier: NCT02051374. First Posted: January 31, 2014. Last Update Posted: February 14, 2018.

## Background

Lumbar degenerative spondylolisthesis (LDS) is the forward slip of one vertebra over another caused by degeneration and instability of facet joints, and degeneration of ligaments and intervertebral discs [1]. Most patients suffer from symptoms related to a concomitant spinal stenosis, such as back pain, radiating pain to the lower extremities, and, typically, increased pain when walking upright and decreased pain when bending forward [2, 3].

Several meta-analyses and systematic reviews have been published with the purpose of providing guidelines on how to surgically treat patients with degenerative spondylolisthesis. Based largely on a pseudorandomized study from 1991 [4], they conclude that there is moderate evidence for a tendency towards better outcome when decompression is combined with fusion [3, 5–7]. A recently published randomized controlled trial (RCT) has lent support to this evidence [8]. However, several cohort studies [9–11] and another recently published RCT [12], have introduced evidence against additional fusion when operating for LDS.

The current evidence cannot support any definite advice on operation method [13–16]. Although challenging, it is important to investigate how to treat this patient group.

### Objectives

#### Primary objective

The primary objective is to detect whether the intervention-related difference in outcome between decompression alone (DA) and decompression with an additional instrumented fusion (DF) 2 years after surgery, is large enough to justify the use of instrumentation. Our hypothesis is that DA is “as good as” DF for the treatment of spinal stenosis with degenerative spondylolisthesis.

#### Secondary objectives

- Health economic analysis: To compare the cost- utility of the investigated treatments DA and DF [17].
- Predictor analysis: To evaluate whether radiological parameters and patient characteristics in the future can be used by clinicians to choose between DA and DF.
- Long-time follow-up studies: The analyses performed at 2-year follow-up will be repeated at 5- and 10-year follow-up.

### Trial design

The proposed trial is a 1:1 block-randomized, controlled, multicenter, non-inferiority trial, with two parallel groups.

The study is one of three trials in the NORDSTEN study, a Norwegian multicenter study on patients with lumbar spinal stenosis [18].

## Methods

The SPIRIT checklist [19] has been used as a template for the present protocol. One exclusion criterion has been detached from the original study protocol (Version 1.0) received January 10, 2014 in Clinicaltrials.gov (Identifier: NCT02051374), see ‘Amendment’.

The report of the trial will be based on an adapted Consolidated Standards of Reporting Trials (CONSORT) checklist for reporting non-inferiority trials [20].

### Participants

The surgeons at the 15 participating hospitals (Table 1) are following the inclusion and exclusion criteria to decide whether a patient is eligible or not.

**Table 1:** Recruiting hospitals

Oslo University Hospital, Orthopedic dept.	
Akershus University Hospital, Orthopedic dept.	
Bærum Hospital, Orthopedic dept.	
Skien Hospital, Orthopedic dept.	
Arendal Hospital, Orthopedic dept.	
Gjøvik Hospital, Orthopedic dept.	
Lillehammer Hospital, Orthopedic dept.	
Stavanger University Hospital, Orthopedic dept. and dept. for Neurosurgery	
Haukeland University Hospital, Orthopedic dept. and dept. for Neurosurgery	
Kysthospitalet i Hagevik, Haukeland University Hospital, Orthopedic dept.	
Ålesund Hospital, Orthopedic dept.	
St. Olav University Hospital, dept. for Neurosurgery	
University Hospital of Northern Norway, dept. for Neurosurgery	
Kristiansand Hospital, Orthopedic dept.	
Elverum Hospital, Orthopedic dept.	

The patients are given verbal and written information about the study and the alternative treatment options. If willing to participate, the patients sign an informed consent form. If a patient does not want to participate in the study, he/she will not be included in the study and will receive normal care and be treated following the hospital’s established procedures. Criteria for inclusion and exclusion are given in Table 2.

**Table 2:** Criteria for inclusion and exclusion for the NORDSTEN/DS trial

Inclusion criteria:	Exclusion criteria:
To be eligible for the study the participants must:	The participants will be excluded from the study if they:
Be over 18 years of age. Understand Norwegian language, spoken and written. Have a spondylolisthesis, with a slip > = 3 mm, verified on standing plain x-rays in lateral view. Have a spinal stenosis in the level of spondylolisthesis, shown on MRI, CT scan or myelogram. Have clinical symptoms of spinal stenosis as neurogenic claudication or radiating pain into the lower limbs, not responding to at least 3 months of qualified conservative treatment. Be able to give informed consent and to respond to the questionnaires.	Are not willing to give written consent. Are participating in another clinical trial that may interfere with this trial. Are ASA- grade > 3. Are older than 80 years. Are not able to fully comply with the protocol, including treatment, follow-up or study procedures (psychosocially, mentally and physically). Have cauda equina syndrome (bowel or bladder dysfunction) or fixed complete motor deficit. Have a slip > = 3 mm in more than one level. Have an isthmic defect in pars interarticularis. Have a fracture or former fusion of the thoracolumbal region. Have had previous surgery in the level of spondylolisthesis. Have a lumbosacral scoliosis of more than 20 degrees verified on AP-view. Have distinct symptoms in one or both legs due to other diseases, e.g. polynevropathy, vascular claudication or osteoarthtritis. Have radicular pain due to a MRI-verified foraminal stenosis in the slipped level, with deformation of the nerve root because of a bony narrowing in the vertical direction.

*MRI* Magnetic resonance imaging, *CT* Computed tomography, *AP* anterior- posterior, *ASA* American Society of Anesthesiologists

**Fig. 1:**
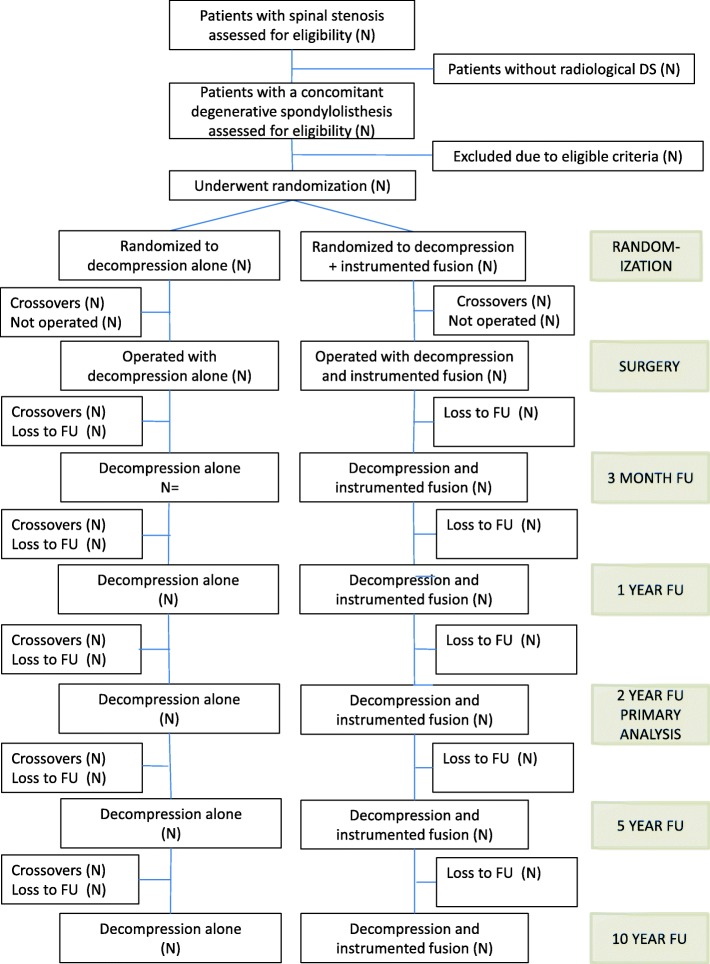
Flow-chart for NORDSTEN-DS. Legend: Eligibility, randomization, treatment and follow-up

All eligible patients are being registered, and the reasons that some are not included are being documented and interpreted. A CONSORT flow chart is illustrated in Fig. 1.

### Interventions

#### Decompression alone

Posterior approach with decompression after microsurgical principles will be performed, and the midline structures will be preserved. The surgeons will either use a microscope or magnifying glasses.

#### Decompression and instrumental fusion

Posterior approach with decompression will be performed, followed by posterolateral pedicle screw fixation with or without an additional cage. The surgeons will either use a microscope or magnifying glasses.

Both groups will receive perioperative intravenous antibiotic prophylaxis. Postoperative care and mobilization will follow each hospital’s normal practices and routines.

### Outcomes

Patient reported outcome measures (PROMs) will be collected preoperatively and at 3 months, 12 months, 2 years, 5 years and 10 years postoperatively. Primary endpoint is at 2-year follow-up. To evaluate the long-term results (5- and 10-year follow-up) we will use the same primary and secondary outcome measurements as at 2-year follow-up. The time schedule for collection of data is shown in Table 3.

**Table 3:** Time schedule for collection of data for the NORDSTEN/DS trial

	Before operation	Hospital stay	3 months	12 months	2 years	5 years	10 years
X-rays	x		x		x	x	x
MRI scan	x						
CT scan					x		
Demographics	x						
Lifestyles	x						
PROMs	x		x	x	x	x	x
Operation data		x					
Data from hospital stay		x					
Complications, and reoperations		x	x	x	x	x	x

*MRI* Magnetic resonance imaging, *CT* Computed tomography, *PROMs* Patient reported outcome measures

The primary outcome is the proportion of responders assessed by the Oswestry Disability Index (ODI) V.2.0 [21, 22]. ODI scores range from 0 to 100, where 100 represent the greatest impairment. Based on former studies [23, 24] and a presently not submitted study from The Norwegian Registry for Spine Surgery (NORSpine), an individual ODI improvement of 30% or more from baseline to follow-up has been chosen as the cut-off for being a responder. Mean scores at follow-up and mean change scores from baseline to follow-up for the ODI scores will be secondary outcomes.

Other secondary outcome measurements are the mean scores at follow-up, the mean changes from baseline to follow-up and the responder rates assessed by the Zürich Claudication Questionnaire [25] [ZCQ; which ranges from 1 to 4 (worst disability)], and by the Numeric Rating Scale for back and leg pain (NRS; which ranges from 0 to 10 (worst pain imaginable)]. Cut-off values for being a responder for ZCQ are defined by Tully et al. [25]. Based on data from NORSpine, the individual thresholds for being a responder are defined as a 40% reduction in the NRS leg pain and a 33% reduction in NRS back pain.

Additional secondary PROMs are the mean scores on the Euroqol 5-D [26] (EQ-5D; which ranges from − 0.6 to 1, with higher scores indicating better quality of life) and the scores on the Global Perceived Effect scale [27] (GPE; a global assessment of patient-rated satisfaction with treatment outcome, with the answers ‘completely recovered’, ‘much improved’, ‘slightly improved’, ‘unchanged’, ‘slightly worse’, ‘much worse’ and ‘worse than ever’). For comparing the failure rate between the groups, the proportion of patients replying ‘much worse’ or ‘worse than ever’ on the GPE scale will be calculated and compared between the groups.

In addition, we will compare the rates of complications and adverse effects (Table 4), the volume of blood loss, the use of blood transfusion perioperatively and postoperatively, the duration of the surgeries from the skin being opened to when it is closed, and the length of hospital stays. Any new surgery in the lumbosacral column from the time of the index operation to follow-up will be recorded and the reoperation rates will be compared. We will distinguish between an operation at the same level as the primary operation and an operation in a new segment.

**Table 4:** Complications and side effects registered during the hospital stay

Perioperative	Postoperative
Dural tear	Liquor leakage
Nerve root lesion	Superficial infection
Operated on the wrong side	Neurological deterioration
Operated on the wrong level	Hematoma requiring reoperation
Amount of bleeding	Use of blood transfusion
Cardiopulmonary complications	Deep infection
Anaphylactic reaction	Thromboembolic episode
Death	Cardiopulmonary complication
Other	Urological complication
	Wrong level/side revealed postoperatively
	Death
	Other

For descriptive interpretation, and for the predictor analyses, The Hopkins symptom check list (HSCL-25; a self-reported questionnaire for assessment of psychological variables) [28], data concerning age, gender, education, work, smoking habits, comorbidity, osteoporosis, the American Society of Anesthesiologists (ASA) grade and prior history of spinal surgery will be recorded preoperatively. For radiological evaluations we will assess the grade of spinal stenosis [29], the foraminal stenosis [30], the magnitude of the olisthesis [31], the segmental instability [31], the orientation of the facet joint [32], the amount of facet joint fluid [33], the degree of disc degeneration [34, 35], the disc height in the level of listhesis [36], the lumbar lordosis [37] and the pelvic parameters (the sacral slope, the pelvic tilt and the pelvic incidence) [37]. A CT scan will be performed at the 2 year follow up for assessment of fusion for the DF group [38]. The time schedule for radiological examinations is given in Table 3. The radiological evaluations will be performed by at least one spine surgeon and one radiologist.

### Sample size

The sample size calculation for efficacy is based on the hypothesis that the 2-year results for the decompression alone group will be at least as good as those from the fusion group when comparing the proportions of responders in each group. The sample size is computed by using the Blackwelder methodology [39]. Based on data from the Norwegian Spine Register, the proportion of responders for the whole treatment group is expected to be 0.70. Choosing a type 1 error = 0.05, power = 0.80 and non-inferiority limit (δ) = 0.15 gives a sample size of 116. Considering these assumptions and adding 10% for possible dropouts, a total of 128 patients are required in each group.

### Recruitment

To ensure a standardized system of enrollment, one or two research coordinators at each hospital manage the practical details regarding registration, collection and further submission of patient data to the central coordinator at the Section for musculoskeletal research (FORMI), Division of neuroscience, at Oslo University Hospital.

### Allocation

The computer generated 1:1 randomization is block-permuted and center-stratified. After the patient has signed the informed consent form, the randomization is performed within the 6 weeks before treatment. The computer generated randomization procedure is concealed and administered by the central coordinator at FORMI, and communicated by phone and by email to the local research coordinator. The coordinator documents the result of the randomization in the patient’s records and assigns the allocated surgical procedure to the surgeon in charge. The randomization process cannot be influenced by the patients, the investigators, the surgeons or any other persons involved in the study.

### Blinding

The treatment given is not blinded for the patients. For analysis and testing of the efficacy variables, the statistician will be blinded for treatment adherence.

### Data collection

The study coordinators are responsible for the collection and administration of data at baseline and at 3-month follow-up. Data from 12-month 2-year, 5-year and 10-year follow-up is collected by the central coordinator at FORMI. All data will be stored at the Faculty of Research support, University of Oslo. The data will be inaccessible to the research group until the first analysis at 2-year follow-up.

### Statistical methods

The first analyses will be performed 2 years after surgery. Long-term follow-up analyses will be performed at 5 and 10 years after surgery.

For the primary objective, the proportion of patients with a reduction in ODI of 30% or more from baseline to 2-year follow-up (responder-rate) is defined as the primary outcome [23, 40]. The null hypothesis (H0) is that the responder rate in the decompression alone group is inferior the responder rate in the decompression and fusion group with an amount of 0.15. H0 will be tested by forming a 95% confidence interval (CI) for the difference of proportions, and H0 will be rejected if the upper limit of the confidence interval (CI) is less than 0.15.

**Fig. 2:**
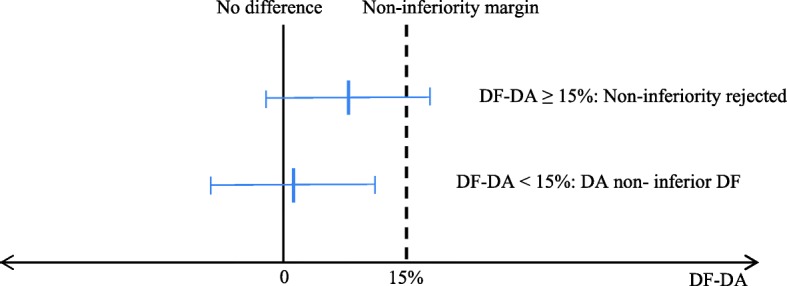
Test for non-inferiority. Legend: The figure shows two alternative results for the primary outcome. DA and DF indicate the proportion of responders in the decompression alone group and decompression plus instrumented fusion group, respectively. The bars indicate the absolute difference in proportion of responders (DF-DA) with 95% confidence interval (CI) limits. Non-inferiority for DA is shown if the upper limit of the 95% CI for the difference is less than 15%

The alternative hypothesis is that the responder rate in the DA group is non- inferior the responder rate in the DF group (Fig. 2).

We have predefined the non- inferior margin to be 0.15 of 1.0, i.e., a 15 percentage difference in the responder rate [41]. With this margin it will be necessary to treat 7 patients or more with fusion in addition to decompression in order to prevent one responder. (Number needed to treat = 1.0:0.15 = 6.67) [42].

The statistical analysis will be done according to intention-to-treat principles (ITT). A sensitivity analysis will be conducted where patient’s crossing over from one treatment to another will receive the last score before crossover. To recommend DA, both the ITT and the sensitivity analysis are required to show non-inferiority.

Descriptive statistics, including measures of centrality and variability, will be used to describe the baseline characteristics of the two treatment groups.

The difference in the proportions of responders (the primary outcome) will be estimated with the Newcombe hybrid score CI [43]. Categorical secondary outcomes will be analyzed with Fisher mid-P tests and Newcombe hybrid score intervals. The GPE responses will be analyzed with a proportional odds logistic regression model. We will use linear mixed models to estimate the difference between the treatment groups for the continuous secondary outcomes (all follow-up measurements from inclusion to 2-year follow-up will be included). Because most change from baseline is expected to occur the first three months, the time development in the linear mixed models will be modelled as piecewise linear, with a knot at 3 months. The models will include fixed effects for treatment group, time, and treatment group x time interaction. A random intercept will be used, and – if possible – a random effect for treatment group.

#### Missing data

For the primary outcome, the primary analysis will be a complete case analysis. If there are patients with missing data in the primary outcome, sensitivity analyses with different imputation scenarios will be performed. The scenarios include all DA patients (with missing data) are responders and none of the DF patients (with missing data) are responders, and vice versa; all DA and all DF patients are responders; all DA and all DF patients are non-responders. Missing data for the continuous secondary outcomes will be handled by the linear mixed models, which include all patients with a measurement at at least one time point.

Complete case analyses will be performed on the categorical secondary outcomes. A significance level of 5% will be used throughout.

#### Analyses of secondary objectives

##### Predictor analysis

The predictor analysis will be performed by use of a pragmatic model-building approach of Hosmer et.al [44]. This method is advocated when risk factor modelling is of interests and not just prediction [45]. Patients treated with decompression alone and decompression with fusion will be analyzed in separate cohorts. For each cohort the following purposefully selected baseline variables will be tested for their association to the primary outcome variable ‘responder’: 1) Patient age; 2) Gender; 3) Comorbidity (ASA group); 4) Body Mass Index; 5) Smoking; 6) ODI score; 7) NRS back pain score; 8) NRS leg pain score; 9) Hopkins symptom check list (HSCL-25); 10) The magnitude of olisthesis; 11) Segmental instability; 12) Presence of foraminal stenosis; 13) Orientation of the facet joint; 14) Amount of facet joint fluid; 15) Disc degeneration; 16) Disc height in the level of olisthesis; 17) Lumbal lordosis; 18) Pelvic incidence.

From a univariate screening, variables with *P* < 0.25 will be included in the multivariate analyses. Since age and gender will be of interests for clinicians when searching for the best choice of treatment, these variables will be included throughout the multivariate analysis. In the second step, the iterative process, covariates are removed if they are non- significant predictors at the 0.1 alpha level and not a confounder. Confounding is defined as a change in any remaining covariate more than 15% when removing a covariate from the model. The covariates will be deleted in descending rang according to the *p*-value. After deleting and refitting, the model will contain only significant covariates and confounders. In the next step, the covariates not selected from the univariate analysis one by one will be tested for their contributions in the presence of variables from the retained model. If significant at alpha level 0.15 they are included for further fitting of the multivariate model. Finally the model is iteratively reduced as before, but only variables additionally added will be excluded. From the final best fitted model for each treatment group, predicted probabilities of being a responder will be estimated for each combination of the covariates. The risk estimates will be used for building matrixes for an individual’s overall risk for being a responder following surgery. Previously, risk matrix models for predicting probability given a set of established predictors has been constructed for other conditions [46, 47].

##### Cost-utility analysis

Cost-utility will be analyzed as the difference in costs between the two treatment groups divided by their difference in Quality adjusted life years (QALYs) gained [48, 49]. QALYs will be estimated by combining EQ-5D index and time, calculating the area under the curve using the trapezoidal method. The results will be presented as an incremental cost-effectiveness ratio (ICER), meaning the cost for each unit of effect (QALY) gained from decompression alone instead of decompression with instrumented fusion. The presentation will be done from a health provider perspective based on data from two-year follow-up.

### Clinical monitoring of the trial

The trial is monitored following the Helsinki Declaration, The International Conference on Harmonisation Guideline for Good Clinical Practice (ICH GCP) [50]. An independent monitor affiliated with Møre and Romsdal Health Trust, without influence on the scientific work, will be responsible for the monitoring. Due to the non-regulated ICH GCP guideline for this trial (not including drug intervention) the risk and safety will be safeguarded at the same level as data quality. All informed consent forms will be checked and all registrations of serious events will be monitored. According to the monitoring plan selected variables will be checked. All hospitals will be visited regularly. Adapted versions of the ‘Investigator’s Site File (ISF)’ and the ‘Trial Master File (TMF)’ will be checked for essential documents during the trial. Queries and deviations will be recorded and reported, and the coordinator at the responsible hospital will have two months to send a written report with the required corrections to the monitor.

### Interim analysis and stopping rules

Due to ethical considerations in agreement with the Norwegian Committee for Medical and Health Research Ethics Midt, an interim analysis for safety will be performed when 75 patients in each group have completed the 12-month follow- up. If one of the proposed stop criteria is fulfilled the study will be terminated:

1. The proportion of patients needing reoperation due to any condition in the operated level(s) is statistically significantly higher in one of the groups.
2. The proportion of responders in the DF group, assessed by the primary outcome measure, is higher than in the DA group by an amount of 0.20.

The interim analysis will be conducted by an independent statistician blinded for treatment adherence. Only data on reoperations and on the primary outcome measure (ODI) will be available to the statistician. The statistician will inform the steering committee, via the central coordinator, whether the study can be continued or not. Further information about the analysis will not be disclosed and will not be available to anyone until the main analysis at 2-year follow-up.

### Ethics and dissemination

The protocol has been approved by the Norwegian Committee for Medical and Health Research Ethics Midt (2013/366).

Storage of data is approved by the Norwegian Data Inspectorate. Written informed consent is obtained from the patients. The project is in accordance with the Helsinki Declaration.

None of the principal investigators have any financial or other competing conflicts of interest.

Trial results will be communicated at national and international conferences and published in well-recognized journals.

## Discussion

The rationale, design and method for this prospective randomized clinical multi-center trial on patients with LDS are presented in the current protocol.

We have chosen a non-inferiority design in order to investigate whether clinical outcomes for decompression alone are not worse than decompression with fusion by more than an acceptable amount. Superiority for decompression alone is not considered to be necessary; it would be an additional benefit [20].

The present study will be the largest powered study comparing decompression alone and decompression with instrumented fusion in a randomized setting. It is designed and powered to provide Level 1 evidence for whether decompression alone can be advocated as the preferred method for surgical treatment of DS or not. We also aim to investigate whether patients can be assigned to the most appropriate surgical method. Finally, results at 5- and 10-year follow-up will provide high level evidence for long-time results for the two methods.

We anticipate enclosing the inclusion by the end of 2017.

## Abbreviations

CT: Computed tomography
DA: Decompression alone
DF: Decompression with an additional fusion
DS: Degenerative Spondylolisthesis
EQ-5D: EuroQol 5-dimensional questionnaire utility index
FU: Follow-up
GPE: Global perceived effect
HSCL-25: Hopkins symptom check list
LSS: Lumbar spinal stenosis
MRI: Magnetic resonance imaging
NNT: Number needed to treat
NRS: Numeric rating scale
ODI: Oswestry disability index
PROMs: Patient reported outcome measures
SAP: Statistical Analysis Plan
ZCQ: Zurich claudication questionnaire –score
